# Time spent in outdoor light is associated with the risk of dementia: a prospective cohort study of 362094 participants

**DOI:** 10.1186/s12916-022-02331-2

**Published:** 2022-04-25

**Authors:** Ling-Zhi Ma, Ya-Hui Ma, Ya-Nan Ou, Shi-Dong Chen, Liu Yang, Qiang Dong, Wei Cheng, Lan Tan, Jin-Tai Yu

**Affiliations:** 1 Department of Neurology, Qingdao Municipal Hospital, Qingdao University, Qingdao, 266071 China; 2 Department of Neurology and Institute of Neurology, Huashan Hospital, State Key Laboratory of Medical Neurobiology and MOE Frontiers Center for Brain Science, Shanghai Medical College, Fudan University, 12th Wulumuqi Zhong Road, Shanghai, 200040 China; 3 National Center for Neurological Disorders, Shanghai, China; 4 Institute of Science and Technology for Brain-Inspired Intelligence, Fudan University, Shanghai, China

**Keywords:** Dementia, Risk factors, Sunlight, Dose-response, UK Biobank

## Abstract

**Background:**

Data on the association between free-living daytime sunlight exposure and incident dementia are scarce. The objective is to evaluate whether the time spent in outdoor light is related to the dementia risk and to investigate whether the optimal duration varies with clinical parameters.

**Methods:**

Data were from a prospective cohort of 362,094 UK Biobank participants. A questionnaire survey was conducted to investigate how many hours the participants spent outdoors on typical summer and winter days. A restricted cubic spline (RCS) was performed to explore the potential nonlinear relationship between sunlight exposure and the risk of dementia. We used multivariate Cox proportional hazard regression models to estimate the hazard ratios (HRs) for the associations between sunlight exposure and dementia outcomes, with the change points as a reference.

**Results:**

After a median follow-up of 9.0 years, 4149 (1.15%) individuals were diagnosed with dementia. RCS showed a J-shaped relationship between time spent in outdoor light and the dementia risk, with the lowest risk at three change points (1.5 h/day on average, 2 h/day in summer, and 1 h/day in winter). Cox hazard regression models showed a marked increase in risk at low exposure (HR=1.287, 95%CI 1.094–1.515) but a relatively slow increase at higher exposure (HR=1.070, 95%CI 1.031–1.10). Results are more pronounced among participants over 60 years old, females, and those with exactly 7 h of sleep every night.

**Conclusions:**

Sunlight exposure had a J-shaped association with dementia risk. Giving detailed guidance on sunlight exposure can effectively prevent dementia.

**Supplementary Information:**

The online version contains supplementary material available at 10.1186/s12916-022-02331-2.

## Background

Sunlight has important effects on vitamin D status, sleep rhythm, and other fields of human physiology; many of which are closely related to dementia [1–3]. As the main source of vitamin D in the human body, exposure to ultraviolet radiation (UV) in sunlight is essential for vitamin D synthesis [2]. Multiple longitudinal cohort studies indicated an association between low circulating vitamin D levels and an increased risk of dementia [2, 4, 5]. Besides, 44% of demented patients have sleep-wake disorders, such as difficulty falling asleep (insomnia), frequent awakening (sleep fragmentation), and nighttime confusion, which are often accompanied by diurnal changes in behavior and cognition [6]. Circadian dysrhythmias have further effects on neuroendocrine systems, including cognitive function and emotion. As the most essential “zeitgeber,” sunlight directly affects wakefulness, mood, and cognition, which is referred to as a non-image-forming (NIF) function [7]. Previous studies have also shown that depression is negatively correlated with sunlight exposure [3]. Patients with depression in a room with eastern light had a mean 3.67-day shorter hospital stay than those in west-facing rooms [8]. It has been proven that less sunlight increases the risk of cognitive decline in patients with depression by 2.58 times [9]. Moreover, bright light treatment (BLT) is considered as a promising non-drug intervention for dementia, since it has positive effects on behavioral and psychological symptoms of dementia, sleep, and circadian rhythms [1].

All those studies provided direct or indirect evidence for the hypothesis that sunlight may affect the risk of dementia. However, few epidemiological studies in the adult population-related these outcomes to daytime sunlight exposure. Previously, little information was available on the effects of natural sunlight on dementia. And as far as we know, the dose-response relationship has not been studied yet [10, 11]. It is worth noting that for individuals aged 4 to 64, there is no UK dietary reference value for vitamin D as they are assumed to get enough vitamin D from daylight exposure alone, which makes our research more meaningful [12–14]. Here, we conducted a large-scale cohort study on the longitudinal correlation between daytime light exposure and dementia-related outcomes in the British population. The aim of our study is to provide guidance on sunlight exposure to effectively prevent dementia in the targeted population living in high latitudes far from the equator.

## Methods

### Study design and participants

UK Biobank is a population-based prospective cohort study that recruited about 500,000 males and females aged 37 to 73 years from 2006 to 2010 [15]. The Ethics Committee permitted the UK Biobank from the Northwest Multi-Center Research Ethics Committee (Research Ethics Committee Reference: 16 /NW/0274). All participants assessed one of 22 assessment centers in England, Scotland, or Wales, covering various settings to provide socio-economic and ethnic heterogeneity and urban-rural mix. This ensures a wide distribution in all exposure ranges to detect the generalized correlation between baseline characteristics and health outcomes. After informed consent, participants accomplished a touch screen questionnaire, oral interview, and physical examination and reserved some biological samples.

### Measurement of sunlight exposure

Participants reported how many hours they respectively spent the daylight outdoors on summer and winter typical days. Data was collected at baseline assessment (2006–2010). This section is defined as fields 1050 and 1060 in the UK Biobank database. Participants described a number using a touch-screen pad or selected integers that included “less than an hour a day,” “do not know,” or “prefer not to answer” in several options set in advance. If participants spent a lot of time outdoors, the average time they spent daily should be provided. Initial data preparation included deleting participants who reported “do not know” or “prefer not to answer” (*n*=33856) and redefining “less than an hour a day” as 0 (*n*=19865). Considering the effective daytime duration in the UK, extreme values larger than the typical day length in summer (16 h, *n*=253) and winter (8 h, *n*=5472) were excluded. To unify the outdoor light exposure time standard, the duration reported in winter and summer was averaged within the participants. We exclude people whose average time is more than twice the standard deviation for analysis. There is a strong correlation between the length of outdoor lighting reported in summer and winter (Pearson’s *r*=0.541, *P*<0.001), indicating that participants with more outdoor light time in summer also tend to spend more time in winter.

### Covariates

The selection of covariates was based on the following criteria: demographic variables, exposure-related variables, and confounding variables associated with AD [16, 17]. The following variables were selected: age, sex, education, skin color, use of sun/UV protection, employment status, sleep duration, and pollution of air, fracture history, vitamin D supplement, hearing loss, smoking status, alcohol use, cardiovascular disease (CVD), total physical activity (TPA), and body mass index (BMI). The response of the skin to UV depends on the difference in skin color caused by the distribution of melanocytes caused by the size, volume, and keratinocytes rather than the difference in the number of melanocytes between races [18, 19]. Thus, we choose skin color rather than race as a covariate. Information on education (with or without a college or university degree), skin color (white or colored), use of sun/UV protection (never/rarely, yes, and do not go out in the sunshine), employment status (yes or no), fracture history in past 5 years (yes or no), vitamin D supplement (yes or no), hearing loss (yes or no), cigarette, and alcohol consumption (never, former, and current) was collected from the touchscreen questionnaire. Sleep duration was divided into short (<7 h per night), normal (7 h per night), and long (>7 h per night) [20]. Considering that high air pollution concentrations induce smog, which attenuates sunlight exposure, we used PM_2.5_ as one of the correction factors. CVD was collected from both the touchscreen questionnaire and verbal interview, including heart attack, angina, stroke, and hypertension. TPA was measured basing the revised International Physical Activity Questionnaire (IPAQ), including the frequency and duration of walking (Field 864 and 874), moderate (Field 884 and 894), and vigorous activity (Field 904 and 914) on a typical day/week over the past 4 weeks [21]. BMI was calculated from weight (kg) and standing height (meters) measured during the medical examination.

### Measurement of outcome

Dementia syndromes were identified from the International Classification of Diseases, 9th and 10th revision (ICD9 and ICD10) codes of hospital inpatient admission data, provided by the UK Hospital Episode Statistics. Corresponding start dates and annual review dates were from Scottish Morbidity Records and Patient Episode Database. Participants followed up on the earliest diagnosis of dementia, the date of death, the date of the last data collection by general practitioners, or the time of the last hospitalization, whichever occurred first. According to the ICD, all-cause dementia was defined as code in ICD-9 codes 290, 290.4, 291.2, 294.1, 331.0-331.2, 331.5, and ICD-10 codes A81.0, F00, F01, F02, F03, F05.1, F10.6, G31.0, G31.1, G31.8, G30, and I67.3 (Additional file 1: Table S1). Additionally, dementia diagnoses were also retrieved from primary care statistical information utilizing reading codes (version 2 [Read v2] and version 3 [CTV3 or Read v3]) [22]. We excluded those who reported “dementia, Alzheimer’s disease, or cognitive impairment” in baseline to reduce the possibility of including prevalent cases in our analyses (*n*=34488). Missing participants without dementia outcomes were excluded (*n*=34572). The missing rate of the cohort is 6.88%.

### Statistical analyses

If the distribution of variables is normal, *t* test was used to compare the average level of the incident dementia group and the no incident dementia group. Otherwise, the Mann-Whitney *U* test was used. Categorical variables were presented as numbers (percentages) and compared by the chi-square test. The dose-response relationship was flexibly modeled by the restricted cubic spline (RCS) with five knots to explore the potential nonlinear correlation between sunlight exposure and the risk of all-cause dementia [23]. With the change points as a reference, univariate and multivariate Cox proportional hazard regression models were fitted to estimate hazard ratios (HRs) and 95% confidence intervals (CIs) for the sunlight exposure on dementia outcomes.

Series sensitivity analyses were conducted to test the robustness of our findings. First, we ran our survival models in white participants to check the potential role of skin color since most participants in this study are white. Second, we excluded the population with dementia events within the first 3 years and a further 10 years of follow-up to avoid potential reverse causation. To assess whether the association between time spent in outdoor light and risk of dementia differed across subpopulations, we examined potential effect modification by age at baseline (<60 and ≥60years old), age of dementia onset (early onset: <65 and late onset: ≥65 years old), and sex (male and female). Considering that limited light can lead to sleep disorders, we performed subgroup analysis according to sleep duration (<7 h, 7 h, and >7 h per night). We used the interaction between sunlight exposure time and each potential modifier to test the homogeneity across stratum-specific HRs.

Statistical analyses were completed using R 3.6.1 (R Foundation, Vienna, Austria). *P* values less than 0.05 were statistically significant.

## Results

### Characteristics of study participants

**Fig. 1:**
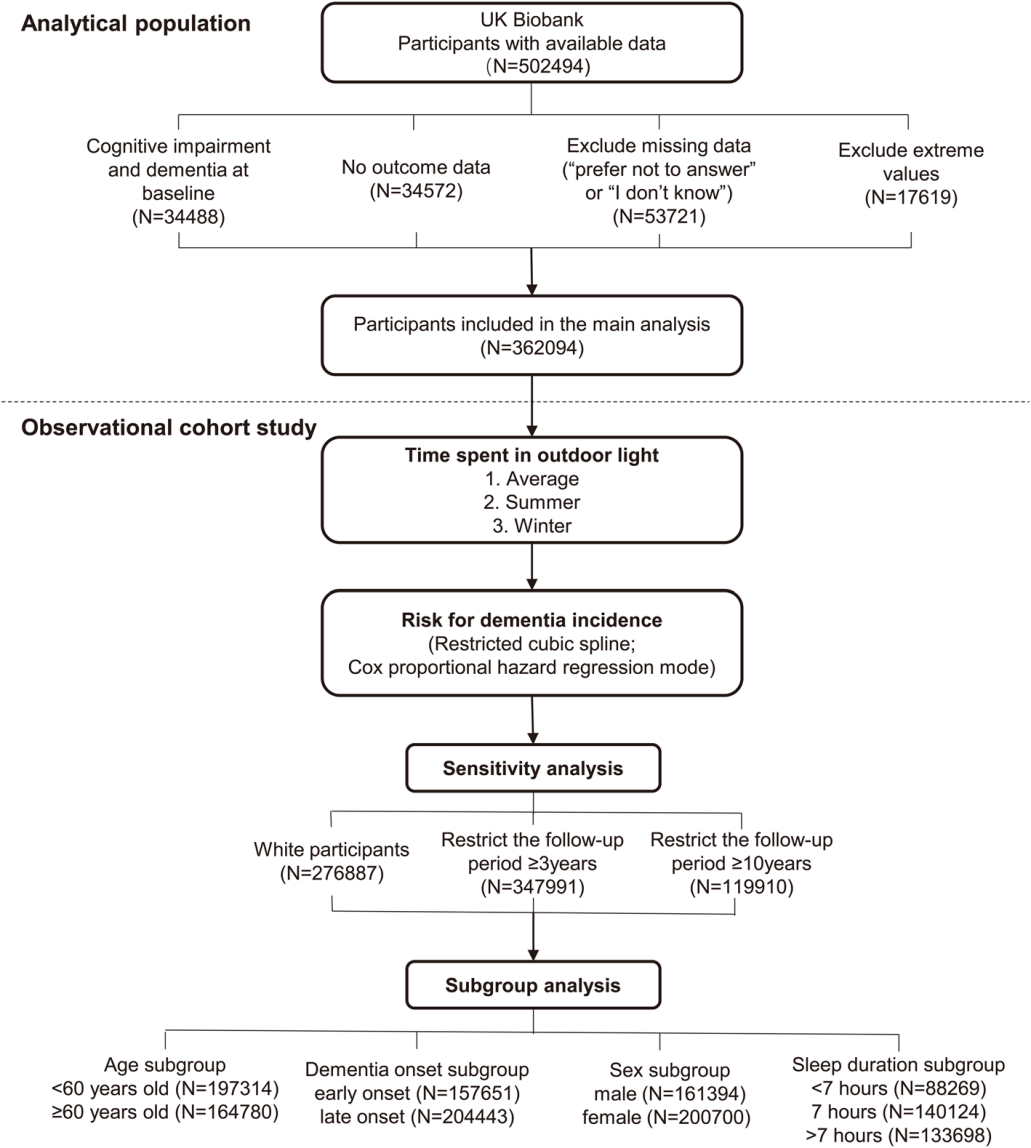
Flow chart of study design

Figure 1 shows the flow chart. A total of 362,094 participants contributing 3,121,329 person-years at risk were included in the primary analyses. Among these participants, 4149 (1.15%) individuals were first-ever diagnosed with dementia during a median follow-up of 9.01 years (IQR 7.0–10.6). The baseline characteristics of the participants were summarized in Table 1 based on whether dementia occurred. Those who converted to dementia were more likely to be older, less educated, never or rarely used sun/UV protection, unemployed, without expected sleep duration, higher PM_2.5_ exposure, smokers, alcohol drinkers, and have more CVD diagnoses (all *P*<0.05). Notably, the average time spent in outdoor light in participants with dementia was significantly longer than those without dementia occurrence (all *P*<0.001).

**Table 1:** Baseline characteristics of study participants by incident dementia status

Variables	Incident dementia	No incident dementia	***P*** value
	(***N***=4149)	(***N***=357945)	
**Age at baseline, years, mean (SD)**	64.49(4.53)	56.87(8.00)	**<0.001**
**Sex, N (%)**			**<0.001**
Male	2211(53.3%)	159183(44.5%)	
Female	1938(46.7%)	198762(55.5%)	
**Education, N (%)**			**<0.001**
≥College or University degree	903(21.8%)	120477(33.7%)	
<College or University degree	1831(44.1%)	178522(49.9%)	
**Skin color, N (%)**			**<0.001**
White	3302(79.6%)	273585(76.4%)	
Colored	847(20.4%)	84352(23.6%)	
**Use of sun/UV protection, N (%)**			**<0.001**
No	625(15.1%)	33268(9.3%)	
Yes	3477(83.8%)	322624(90.1%)	
Do not go out in sunshine 3	45(1.1%)	2038(5.7%)	
**Employment status, N (%)**			**<0.001**
Yes	753(18.1%)	203004(56.7%)	
No	3396(81.9%)	154941(43.3%)	
**Sleep duration, hours, N (%)**			**<0.001**
<7	1070(25.8%)	87199(24.4%)	
7	1246(30.0%)	138878(38.8%)	
>7	1833(44.2%)	131868(36.8%)	
**PM**_**2.5**_**, ug/m**^**3**^**, mean (SD)**	10.04(1.05)	9.97(1.05)	**<0.001**
**Fracture history, N (%)**			0.348
Yes	414(10.0%)	34184(9.6%)	
No	3711(90.0%)	322213(90.4%)	
**Vitamin D supplement, N (%)**			0.092
Yes	177(4.3%)	13490(3.8%)	
No	3944(95.7%)	343441(96.2%)	
**Hearing loss, N (%)**			**<0.001**
Yes	1440(36.1%)	90025(26.1%)	
No	2548(63.9%)	254597(73.9%)	
**Smoking status, N (%)**			**<0.001**
Never	1901 (46.1%)	194259 (54.4%)	
Previous	1805 (43.7%)	126431 (35.4%)	
Current	420 (10.2%)	36256 (10.2%)	
**Alcohol drinker status, N (%)**			**<0.001**
Never	280 (6.8%)	14058 (3.9)	
Previous	295 (7.1%)	12469 (3.5)	
Current	3566 (86.1%)	331202 (92.6)	
**CVD diagnosed by doctor, N (%)**			**<0.001**
Heart disease ^a^	582(14.1%)	16708(4.7%)	
Stroke	243(5.9%)	5503(1.5%)	
Hypertension	1744(42.1%)	98880(27.7%)	
**TPA, MET-mins/week, mean (SD)**	2423.34 (2875.69)	2449.20 (2746.68)	0.555
**BMI, kg/m**^**2**^**, mean (SD)**	27.72(4.95)	27.44(4.80)	**<0.001**
**Time spent in outdoor light, hours/day, mean (SD)**			
Average	2.98(1.51)	2.51(1.45)	**<0.001**
Summer	3.99(2.05)	3.46(1.97)	**<0.001**
Winter	1.97(1.46)	1.57(1.31)	**<0.001**

^a^Heart disease group includes myocardial infarction and angina. Data are n (%) and mean (SD). *P* values are derived using ether Student’s test, Mann-Whitney U test, or Chi-square test

*Abbreviation*: *SD* Standard deviation, *UV* Ultraviolet radiation, *CVD* Cardiovascular disease, *TPA* Total physical activity, *BMI* Body mass index

### Association of time spent in outdoor light with dementia

**Fig. 2:**
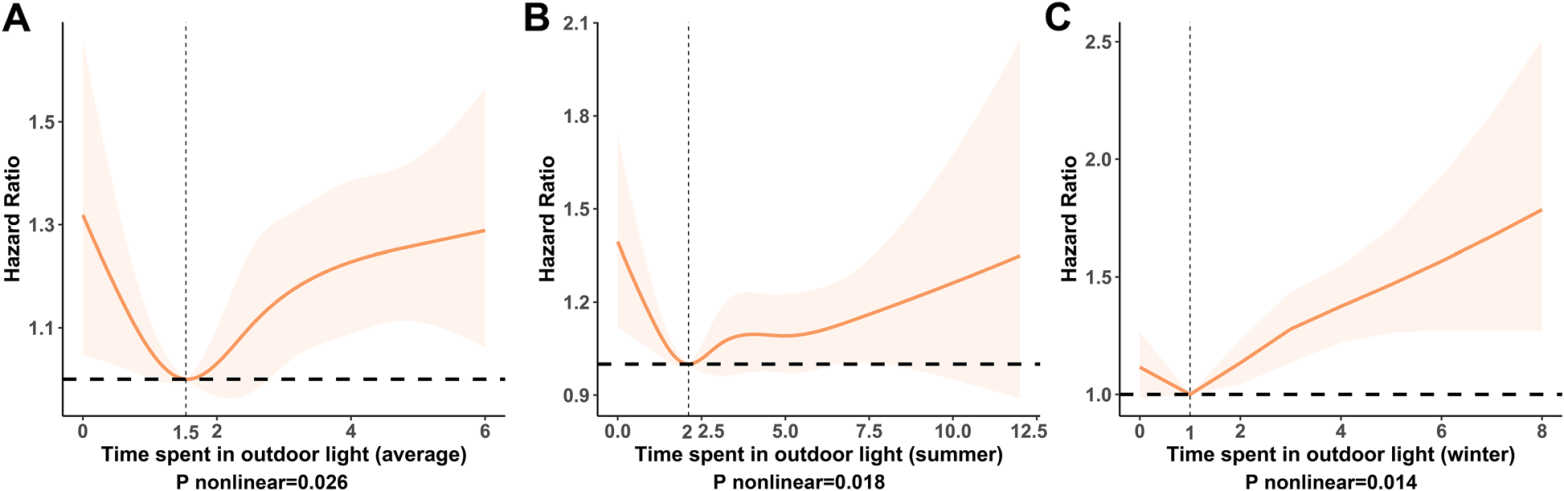
The correlation between outdoor light time and incident dementia during follow-up. The hazard ratio for dementia with the corresponding 95% confidence interval as a function of outdoor light time from Cox proportional hazard regression models adjusted for age, sex, education, skin color, use of sun/UV protection, employment status, sleep duration, PM_2.5_, fracture history, vitamin D supplement, hearing loss, smoking status, alcohol use, CVD, TPA, and BMI. Abbreviation: CVD cardiovascular disease, UV ultraviolet radiation, TPA total physical activity, BMI body mass index

Estimated associations between sunlight exposure (as a continuous variable) and dementia outcome are shown from nonlinear spline models. Associations between time spent in outdoor light and risk of all-cause dementia were nonlinear in average, summer, and winter. Significant increases in risk were observed for dementia at both low and high exposure times (Fig. 2). The nadir for dementia risk was estimated from piecewise linear models to be at an average of 1.5 h/day, summer of 2 h/day, and winter of 1 h/day (Table 2). Multivariable Cox regression analyses showed that compared with participants who received an average of 1.5 h outdoor light per day, participants with less (adjusted HR=1.184 95%CI 1.000–1.401) or more (adjusted HR=1.210, 95%CI 1.054–1.390) time had a higher risk of dementia. Compared with participants who received 2 h outdoor light per day in summer, participants with less (adjusted HR=1.182, 95%CI 1.008–1.387) or more (adjusted HR=1.086, 95%CI 0.973–1.213) time tended to have a higher risk of dementia. This change point is 1 h for winter (less, adjusted HR=1.139, 95%CI 1.001–1.295; more, adjusted HR=1.242, 95%CI 1.129–1.367).

**Table 2:** Risk of incident dementia below and above the change point of sunlight exposure (categorical variable)

Time spent in outdoor light	Number of incident dementia/Number of participants	Model 1 (Age, Sex)		Model 2 (+Education, Skin color, Use of sun/UV Protection, Employment status, Sleep duration, PM2.5)		Model 3 (+Fracture history, Vitamin D supplement, hearing loss, smoking status, alcohol use, CVD, TPA, BMI)	
		HR (95%CI)	***P*** Value	HR (95%CI)	***P*** Value	HR (95%CI)	***P*** Value
**Average**							
1.5 hours	371/49938	reference		reference		reference	
below 1.5 hours	535/72191	1.199 (1.050-1.369)	**0.007**	1.176 (1.002-1.380)	**0.048**	1.184 (1.000-1.401)	**0.049**
above 1.5 hours	3243/239965	1.215 (1.091-1.353)	**<0.001**	1.204 (1.056-1.373)	**0.006**	1.210 (1.054-1.390)	**0.007**
**Summer**							
2 hours	657/82473	reference		reference		reference	
below 2 hours	395/50233	1.213 (1.071-1.375)	**0.002**	1.163 (0.998-1.354)	0.053	1.182 (1.008-1.387)	**0.040**
above 2 hours	3097/229388	1.116 (1.026-1.215)	**0.011**	1.103 (0.994-1.224)	0.065	1.086 (0.973-1.213)	0.140
**Winter**							
1 hours	1099/126309	reference		reference		reference	
below 1 hours	621/73877	1.169 (1.059-1.290)	**0.002**	1.158 (1.025-1.308)	**0.018**	1.139 (1.001-1.295)	**0.048**
above 1 hours	2429/161908	1.235 (1.150-1.327)	**<0.001**	1.246 (1.139-1.363)	**<0.001**	1.242 (1.129-1.367)	**<0.001**

The results are derived from Cox hazard regression models in three models, with the sunlight exposure as a categorical variable. Bold indicates statistical significance (*P* value<0.05)

*Abbreviation*: *HR* Hazard ratio, *CI* Confidence interval, *CVD* Cardiovascular disease, *TPA* Total physical activity, *BMI* Body mass index

More specifically, we prefer to describe this nonlinear relationship with “J shape”. Since for sunlight exposure, a marked increase in risk was observed at low exposure, but a relatively slow elevation in risk at higher exposure (Fig. 2). Table 3 shows the results of the multivariate Cox proportional hazard regression models between sunlight exposure time and dementia as a continuous variable, which proves the above conclusion. Continuous exposure time was positively correlated with poor prognosis. Compared with the reference group (average, 1.5 h/day; summer, 2 h/day; winter, 1 h/day), both low and high sunlight exposure time groups were associated with a higher risk of dementia (average: low, HR=1.287 per 0.5 h, 95%CI 1.094–1.515 vs. high, HR=1.070 per 0.5 h, 95%CI 1.031–1.110); summer: low, HR=1.193 per 1 h, 95%CI 1.069–1.331 vs. high, HR=1.026 per 1 h, 95%CI 1.000–1.052; winter: low, HR=1.184 per 1 h, 95%CI 1.039–1.349 vs. high, HR=1.106 per 1 h, 95%CI 1.066–1.148).

**Table 3:** Risk of incident dementia below and above the change point of sunlight exposure (continuous variable)

Time spent in outdoor light	Model 1 (Age, Sex)		Model 2 (+Education, Skin color, Use of sun/UV protection, Employment status, Sleep duration, PM_**2.5**_)		Model 3 (+Fracture history, Vitamin D supplement, hearing loss, smoking status, alcohol use, CVD, TPA, BMI)	
	HR (95%CI)	***P*** Value	HR (95%CI)	***P*** Value	HR (95%CI)	***P*** Value
**Average**						
1.5 hours	reference		reference		reference	
below 1.5 hours (per 0.5 hour)	1.353 (1.199-1.528)	**<0.001**	1.261 (1.084-1.468)	**0.003**	1.287 (1.094-1.515)	**0.002**
above 1.5 hours (per 0.5 hour)	1.071 (1.044-1.099)	**<0.001**	1.069 (1.033-1.105)	**<0.001**	1.070 (1.031-1.110)	**<0.001**
**Summer**						
2 hours	reference		reference		reference	
below 2 hours (per 1 hour)	1.232 (1.137-1.336)	**<0.001**	1.180 (1.065-1.308)	**0.002**	1.193 (1.069-1.331)	**0.002**
above 2 hours (per 1 hour)	1.032 (1.013-1.051)	**0.001**	1.029 (1.005-1.053)	**0.019**	1.026 (1.000-1.052)	**0.048**
**Winter**						
1 hours	reference		reference		reference	
below 1 hours (per 1 hour)	1.182 (1.071-1.305)	**0.001**	1.173 (1.038-1.326)	**0.010**	1.184 (1.039-1.349)	**0.011**
above 1 hours (per 1 hour)	1.099 (1.071-1.127)	**<0.001**	1.104 (1.066-1.142)	**<0.001**	1.106 (1.066-1.148)	**<0.001**

The results are derived from Cox hazard regression models in three models, with the sunlight exposure as a continuous variable. Bold indicates statistical significance (*P* value<0.05)

*Abbreviation*: *HR* Hazard ratio, *CI* Confidence interval, *CVD* Cardiovascular disease, *TPA* Total physical activity, *BMI* Body mass index

### Additional analyses

Sensitivity analyses did not affect the assessment of the relationship between sunlight exposure time and dementia risk (Additional file 1). Similar results were observed after excluding participants with less than 3 and 10 years of follow-up, respectively (Additional file 1: Figure S2-3 and Table S3-4). The results of the White population showed the conclusion is also applicable to white participants (Additional file 1: Figure S1 and Table S2). To avoid the possible influence of missing data on the results, we repeated our analyses in a subset with complete covariates data (*n*=222479). The results were barely changed (Additional file 1: Figure S4 and Table S5).

Among participants equal to or older than 60 years old, there was a J-shaped relationship between sunlight exposure time and dementia risk, with the bottoms of the spline curve is about 2 h per day. This value is about 3 h/day in summer and about 1 h/day in winter. It is noteworthy that all these time points are longer than studies across all populations. A dose-dependent association between sunlight exposure time and the risk of dementia was not evident among participants under 60 (Additional file 1: Figure S5 and Table S6). The same phenomenon was observed in early and late onset dementia subgroups (Additional file 1: Figure S6 and Table S7). In females, the dementia risk was lowest at around an average of 2 h per day and increased dose-dependent if the time was below or above 2 h per day. A linear dose-response relationship was not observed between sunlight exposure time and the risk of dementia in males (Additional file 1: Figure S7 and Table S8). The subgroup analysis of sleep duration showed that even participants who slept 7 h per night needed sunlight to reduce the risk of dementia, with the bottoms of the spline curve being about 1.5 h per day. This value is 2 h/day in summer and 1 h/day in winter. (Additional file 1: Figure S8 and Table S9).

## Discussion

The present study revealed that sunlight exposure time was associated with the risk of dementia. We observed a J-shaped association between outdoor sunlight exposure time and all-cause dementia risk, with the lowest risk at three change points (2 h/day in summer and 1 h/day in winter, and 1.5 h/day on average). This relationship did not change significantly after adjusting for various confounding factors.

Our findings were mostly consistent with previous evidence. A previous study has shown that a sunny June noon with an average sunshine time of about 105 min is most suitable for Alaska Anchorage to avoid vitamin D deficiency [24]. The latitude of this area (60°N) is like that of the UK. As we know, estimated cumulative UV exposure can be used to characterize an individual’s long-term vitamin D status [24]. Furthermore, the light and temperature models for the associations of light and temperature measurements with stroke subtypes also showed that stroke risk was the lowest in the second quartile of sunlight exposure, and higher or lower exposure increased the risk of stroke [25]. Surprisingly, the most appropriate sunlight exposure duration is shorter in winter than in summer. We speculate that this may be since some diseases are more likely to occur at low temperatures in winter. For example, as a seasonal disease, stroke showed an increased incidence in winter but a decreased incidence in summer [26, 27]. It is noteworthy that under less-than-ideal conditions, such as morning, evening, and cloudy conditions, there is less UV in the environment, and exposure to the same dose of UV may take a longer time.

There might be a variety of mechanisms underlying the relationship between sunlight and cognitive decline. Studies have pointed out that vitamin D receptors are widely present in neurons and glial cells. Vitamin D is involved in a variety of crucial pathways for brain health, involving neurotransmission, neuroprotection, regulation of immune response, inhibition of pro-inflammatory agents, and regulation of oxidative stress [2, 4]. There were evidences that the relationship between serum vitamin D concentration, and disease outcome does not seem to be a simple linear relationship [28, 29]. The data of long-term vitamin D levels in individuals are difficult to obtain. Since the progression of cognitive decline in the elderly is a long period, serum 25-hydroxyvitamin D, which is widely used to indicate vitamin D, can only indicate the temporary vitamin level. In addition, vitamin D levels in the tissues vary significantly with sunlight. Therefore, we used sunlight exposure time to roughly the vitamin D level in the elderly. Prospective studies are a trustworthy method to explore the association between vitamin D and cognitive function, but they take years or even decades. Most vitamin D is synthesized in the skin exposed to UV. The Department of Health and Social Care in the UK constantly rejects the proposal to add vitamin D into food, such as milk, bread, and orange juice [30]. Therefore, it is reasonable to use sunlight exposure to represent an individual’s long-term vitamin D level. In addition to vitamin D status, the environmental sunlight may also affect the physiological and cognitive function of the human body by regulating the circadian rhythm and affecting the suprachiasmatic nuclei (SCN) which are also called the body’s internal clock. One of the regulatory functions of SCN is to inhibit the pineal gland from converting serotonin into melatonin, which is involved in many mental and cognitive disorders [31].

However, excessive sunlight exposure is closely related to an increased risk of many negative consequences for human health, including sunburn, skin cancer (melanoma, lip cancer, and keratinocytes), and eye diseases (cataracts, ultraviolet keratitis). Suberythema UV doses also have biological effects. The absorption of UV by epidermal cells of the skin and eyes leads to the production of reactive oxygen species and nitrogen substances, which can injure biological molecules, such as the membrane lipids and DNA. UV directly damages DNA by forming pyrimidine dimers [32]. It is worth noting that in an upright state, the head of the human body is exposed directly to solar radiation, which may raise brain temperature to exceed core temperature, as broad-spectrum light penetrates several millimeters into the skin and heats the underlying tissues. There is an inverted U-shaped relationship between hyperthermia and cognitive performance. Significant cognitive declines in head and neck exposure tests are similar to that observed during hyperthermia therapy [33]. Long-term exposure of head and neck to sunlight causes a 1 °C increase in core temperature, which will cause worse cognitive performance [34]. Therefore, exploring the optimal sunlight exposure time can help us get more benefits and less damage.

The relationship between sunlight exposure and cognitive decline was more robust in females and older participants. The above gender tendency and age tendency are consistent with previous studies on the relationship between temperature and mortality, which found stronger associations in females and elders [35, 36]. Some reasons may explain our findings. Women are more likely to be affected by weather, and their clothes typically expose more skin. And elders are more likely to be affected by the changes during the aging process, such as cardiovascular events and the unignorable cumulative effect of light [37]. At the same time, the cumulative effect of light cannot be ignored. Besides, we found that elders seem to need a relatively longer duration of sunlight exposure to achieve the protective effect. The reason for this phenomenon may be that with increasing age, the amount of light reaching the retina decreases due to the yellowing of the lens and the contraction of the pupils. Although the compensation mechanism may maintain photosensitivity to some extent, yellowing is related to sleep disorders in the elderly [38, 39]. We found that even for participants who slept an optimal 7 h per night, sunlight exposure influenced the risk of dementia. Therefore, a 7-h sleep is not enough to offset the risk, and it needs to cooperate with the management of sunlight exposure.

We are the first to explore the nonlinear relationship between sunlight exposure time and the risk of dementia with a large cohort of cognitively intact participants. However, there are still some limitations. First, our research scheme reflects a potential sunlight mode. More details remain to be explored because the UK Biobank database did not collect the specific period of subjects receiving illumination. The use of rigorous design and detailed intervention characteristics reports, such as illuminance and temperature are essential for a prevention strategy. Second, differences in duration and UV intensity at different latitudes suggest that our conclusions may be hard to apply to all countries and regions. It needs further research on the specific optimal duration for other areas. Third, the duration of sunlight exposure is self-reported, which may lead to inaccurate responses, although most large-scale epidemiological studies rely on self-reported questionnaires. Fourth, bias due to unmeasured confounders persisted despite multiple corrections. Finally, the participants were predominantly white, with a slightly higher proportion of the affluent group. Further research are needed to investigate the extent to which these findings could be generalized to other populations.

## Conclusions

In conclusion, there is a J-shaped correlation between outdoor sunlight exposure time and dementia risk. Detailed guidance on sunlight exposure can effectively prevent dementia in British people living in high latitudes far from the equator.

## Supplementary Information

**Additional file 1: Figure S1.** The correlation between sunlight exposure time and incident dementia during follow-up in white participants. **Figure S2.** The correlation between sunlight exposure time and incident dementia during follow-up excluding participants less than three years of follow-up. **Figure S3.** The correlation between sunlight exposure time and incident dementia during follow-up excluding participants less than ten years of follow-up. **Figure S4.** The correlation between sunlight exposure time and incident dementia during follow-up in the subgroup with complete covariates data (*n*=222479). **Figure S5.** The correlation between sunlight exposure time and incident dementia during follow-up in age subgroups. **Figure S6.** The correlation between sunlight exposure time and incident dementia during follow-up in onset age subgroups. **Figure S7.** The correlation between sunlight exposure time and incident dementia during follow-up in sex subgroups. **Figure S8.** The correlation between sunlight exposure time and incident dementia during follow-up in sleep duration subgroups. **Table S1.** UKB field ID for dementia outcome used in the paper. **Table S2.** Risk of incident dementia below and above the change point of sunlight exposure in white participants. **Table S3.** Risk of incident dementia below and above the change point of sunlight exposure excluding participants less than three years of follow-up. **Table S4.** Risk of incident dementia below and above the change point of sunlight exposure excluding participants less than ten years of follow-up. **Table S5.** Risk of incident dementia below and above the change point of sunlight exposure in the subgroup with complete covariates data (*n*=222479). **Table S6.** Risk of incident dementia below and above the change point of sunlight exposure in subgroups of age. **Table S7.** Risk of incident dementia below and above the change point of sunlight exposure in subgroups of onset age. **Table S8.** Risk of incident dementia below and above the change point of sunlight exposure in sex subgroups. **Table S9.** Risk of incident dementia below and above the change point of sunlight exposure in subgroups of sleep duration.

## Data Availability

The data that support the findings of this study are available from the UK Biobank project site, subject to registration and application process. Further details can be found at https://www.ukbiobank.ac.uk.

## Abbreviations

UV: Ultraviolet radiation
NIF: Non-image-forming
BLT: Bright light treatment
CVD: Cardiovascular disease
TPA: Total physical activity
BMI: Body mass index
IPAQ: International Physical Activity Questionnaire
ICD: International Classification of Diseases
RCS: Restricted cubic spline
HR: Hazard ratio
CI: Confidence interval
SCN: Superchiasmatic nuclei
