# Supplementary material for: Time spent in outdoor light is associated with the risk of dementia: a prospective cohort study of 362094 participants
Source: BMC Med. 2022 Apr 25;20:132. doi: 10.1186/s12916-022-02331-2 (PMC9036798; doi:10.1186/s12916-022-02331-2)
Supplement: Supplementary file 1 — Additional file 1: Figure S1. The correlation between sunlight exposure time and incident dementia during follow-up in white participants. Figure S2. The correlation between sunlight exposure time and incident dementia during follow-up excluding participants less than three years of follow-up. Figure S3. The correlation between sunlight exposure time and incident dementia during follow-up excluding participants less than ten years of follow-up. Figure S4. The correlation between sunlight exposure time and incident dementia during follow-up in the subgroup with complete covariates data (n=222479). Figure S5. The correlation between sunlight exposure time and incident dementia during follow-up in age subgroups. Figure S6. The correlation between sunlight exposure time and incident dementia during follow-up in onset age subgroups. Figure S7. The correlation between sunlight exposure time and incident dementia during follow-up in sex subgroups. Figure S8. The correlation between sunlight exposure time and incident dementia during follow-up in sleep duration subgroups. Table S1. UKB field ID for dementia outcome used in the paper. Table S2. Risk of incident dementia below and above the change point of sunlight exposure in white participants. Table S3. Risk of incident dementia below and above the change point of sunlight exposure excluding participants less than three years of follow-up. Table S4. Risk of incident dementia below and above the change point of sunlight exposure excluding participants less than ten years of follow-up. Table S5. Risk of incident dementia below and above the change point of sunlight exposure in the subgroup with complete covariates data (n=222479). Table S6. Risk of incident dementia below and above the change point of sunlight exposure in subgroups of age. Table S7. Risk of incident dementia below and above the change point of sunlight exposure in subgroups of onset age. Table S8. Risk of incident dementia below and above the change point of [file 12916_2022_2331_MOESM1_ESM.docx]

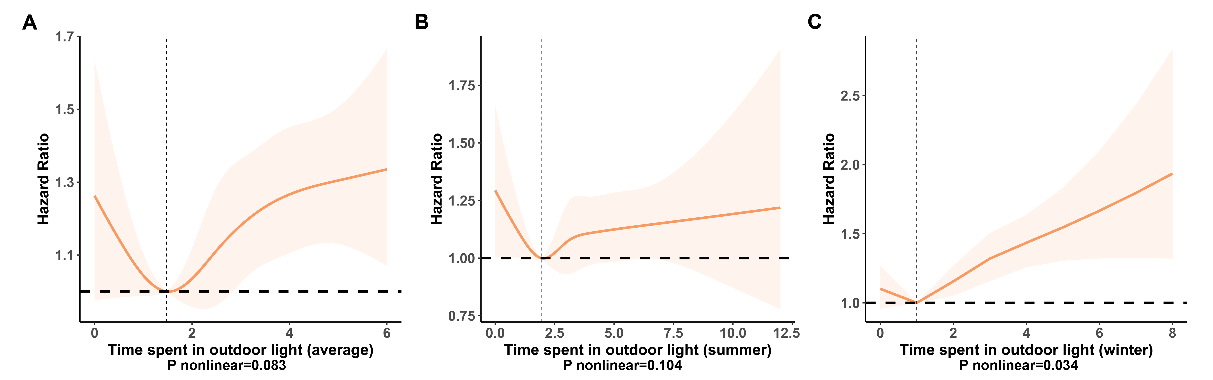


Figure S1. The correlation between sunlight exposure time and incident dementia during follow-up in white participants. The hazard ratio from Cox proportional hazard regression models adjusted for age, sex, education, use of sun/UV protection, employment status, sleep duration, PM_2.5_, fracture history, Vitamin D supplement, hearing loss, smoking status, alcohol use, CVD, TPA, and BMI.

Abbreviation: UV, Ultraviolet radiation; CVD, Cardiovascular disease; TPA, Total physical activity; BMI, Body mass index.


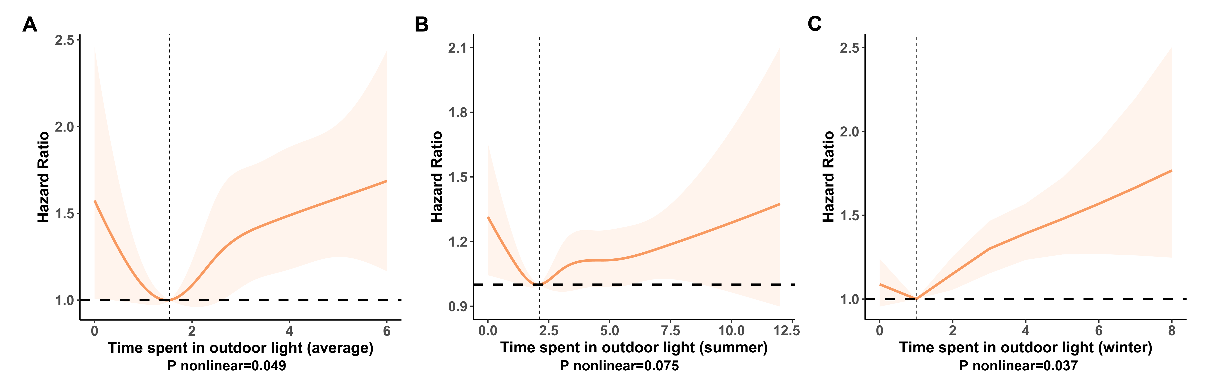


Figure S2. The correlation between sunlight exposure time and incident dementia during follow-up excluding participants less than three years of follow-up. The hazard ratio from Cox proportional hazard regression models adjusted for age, sex, education, use of sun/UV protection, employment status, sleep duration, PM_2.5_, fracture history, Vitamin D supplement, hearing loss, smoking status, alcohol use, CVD, TPA, and BMI.

Abbreviation: UV, Ultraviolet radiation; CVD, Cardiovascular disease; TPA, Total physical activity; BMI, Body mass index.


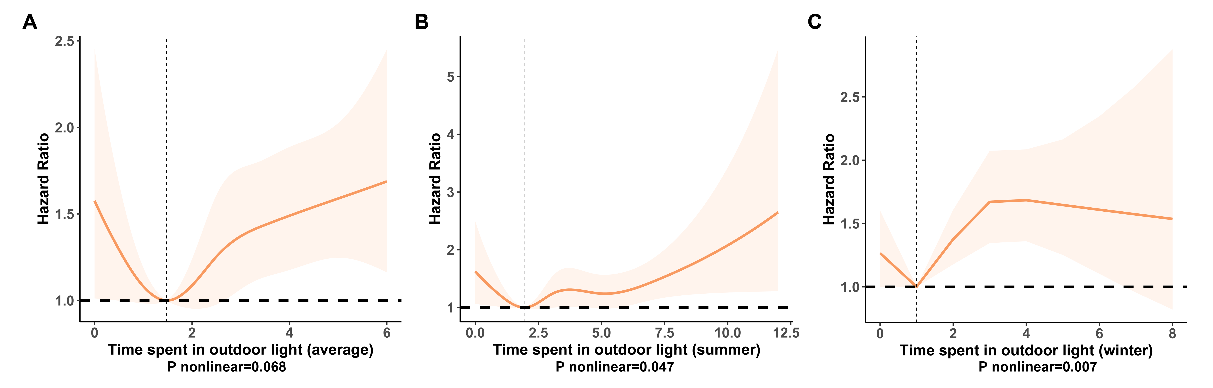


Figure S3. The correlation between sunlight exposure time and incident dementia during follow-up excluding participants less than ten years of follow-up. The hazard ratio from Cox proportional hazard regression models adjusted for age, sex, education, use of sun/UV protection, employment status, sleep duration, PM_2.5_, fracture history, Vitamin D supplement, hearing loss, smoking status, alcohol use, CVD, TPA, and BMI.

Abbreviation: UV, Ultraviolet radiation; CVD, Cardiovascular disease; TPA, Total physical activity; BMI, Body mass index.


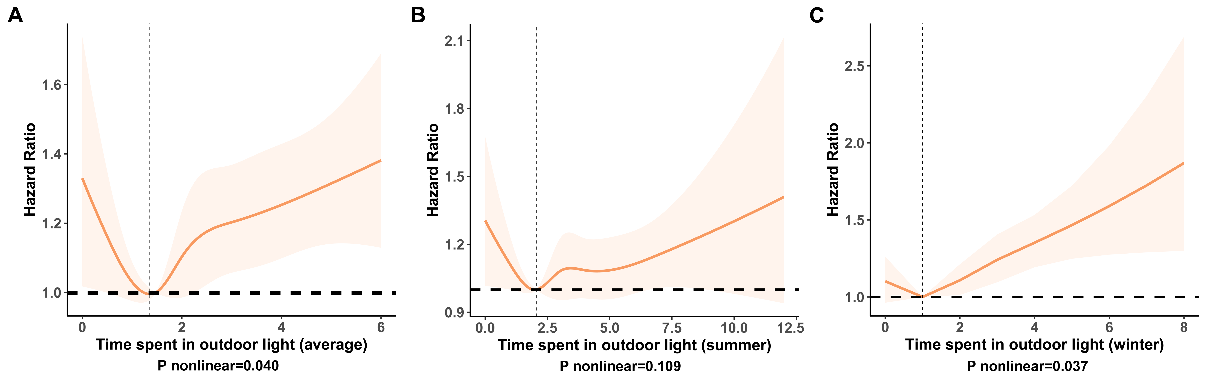


Figure S4. The correlation between sunlight exposure time and incident dementia during follow-up in subgroup with complete covariates data (n=222479). The hazard ratio from Cox proportional hazard regression models adjusted for age, sex, education, use of sun/UV protection, employment status, sleep duration, PM_2.5_, fracture history, Vitamin D supplement, hearing loss, smoking status, alcohol use, CVD, TPA, and BMI.

Abbreviation: UV, Ultraviolet radiation; CVD, Cardiovascular disease; TPA, Total physical activity; BMI, Body mass index.


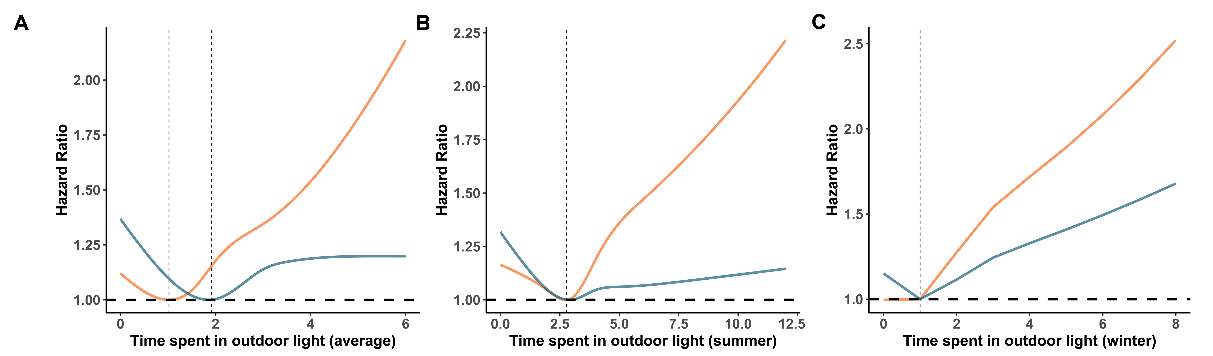


Figure S5. The correlation between sunlight exposure time and incident dementia during follow-up in age subgroups. Yellow lines represent subgroups younger than 60 years and blue lines represent subgroups older than or equal to 60 years. The hazard ratio from Cox proportional hazard regression models adjusted for age, sex, education, use of sun/UV protection, employment status, sleep duration, PM_2.5_, fracture history, Vitamin D supplement, hearing loss, smoking status, alcohol use, CVD, TPA, and BMI.

Abbreviation: UV, Ultraviolet radiation; CVD, Cardiovascular disease; TPA, Total physical activity; BMI, Body mass index.


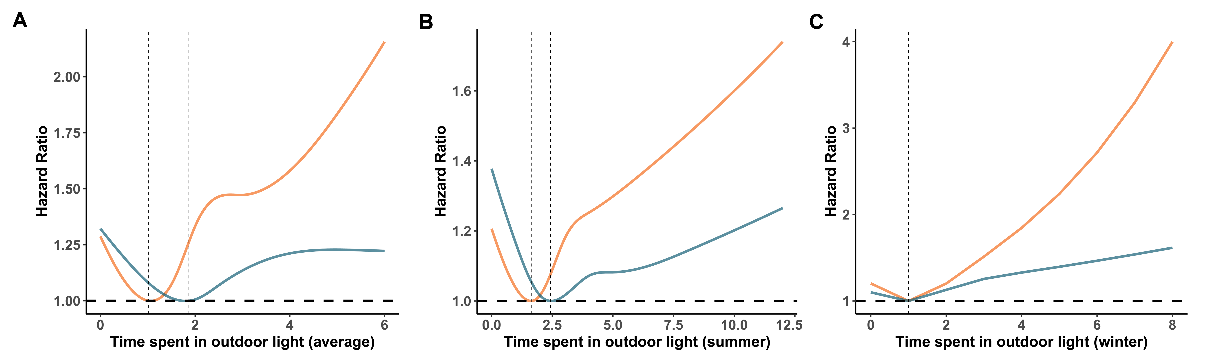


Figure S6. The correlation between sunlight exposure time and incident dementia during follow-up in onset age subgroups. Yellow lines represent subgroups younger than 65 years and blue lines represent subgroups older than or equal to 65 years. The hazard ratio from Cox proportional hazard regression models adjusted for age, sex, education, use of sun/UV protection, employment status, sleep duration, PM2.5, fracture history, Vitamin D supplement, hearing loss, smoking status, alcohol use, CVD, TPA, and BMI.

Abbreviation: UV, Ultraviolet radiation; CVD, Cardiovascular disease; TPA, Total physical activity; BMI, Body mass index.


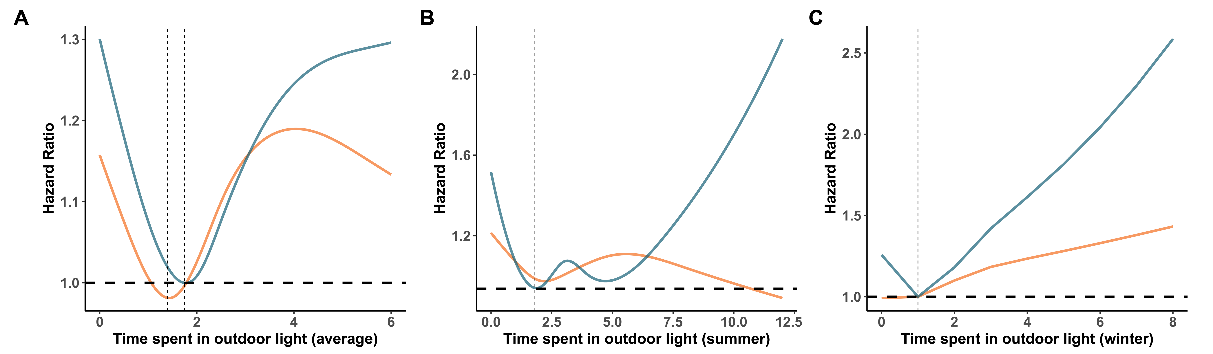


Figure S7. The correlation between sunlight exposure time and incident dementia during follow-up in sex subgroups. Yellow lines represent male subgroup and blue lines represent female subgroup. The hazard ratio from Cox proportional hazard regression models adjusted for age, education, use of sun/UV protection, employment status, sleep duration, PM2.5, fracture history, Vitamin D supplement, hearing loss, smoking status, alcohol use, CVD, TPA, and BMI.

Abbreviation: UV, Ultraviolet radiation; CVD, Cardiovascular disease; TPA, Total physical activity; BMI, Body mass index.


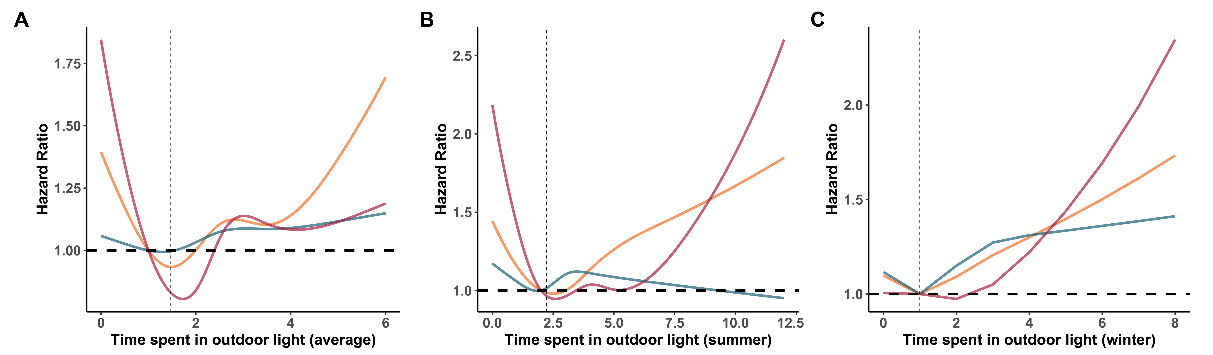


Figure S8. The correlation between sunlight exposure time and incident dementia during follow-up in sleep duration subgroups. Yellow lines represent the subgroup whose sleep duration is less than 7 hours, blue lines represent the subgroup whose sleep time is 7 hours, and the purple lines represent the subgroup whose sleep time is more than 7 hours. The hazard ratio from Cox proportional hazard regression models adjusted for age, sex, education, use of sun/UV protection, employment status, PM2.5, fracture history, Vitamin D supplement, hearing loss, smoking status, alcohol use, CVD, TPA, and BMI.

Abbreviation: UV, Ultraviolet radiation; CVD, Cardiovascular disease; TPA, Total physical activity; BMI, Body mass index.

Table S1. UKB field ID for dementia outcome used in the paper.

| **Field ID** | **Annotation** |
| --- | --- |
| ICD-9 290 | Senile and presenile organic psychotic conditions |
| ICD-9 290.4 | Arteriosclerotic dementia |
| ICD-9 291.2 | Other alcoholic dementia |
| ICD-9 294.1 | Dementia in conditions classified elsewhere |
| ICD-9 331.0 | Alzheimer’s disease |
| ICD-9 331.1 | Pick’s disease |
| ICD-9 331.2 | Senile degeneration of brain |
| ICD-9 331.5 | Jakob-creutzfeldt disease |
| ICD-10 A81.0 | Creutzfeldt-Jakob disease |
| ICD-10 F00 | Dementia in Alzheimer's disease |
| ICD-10 F01 | Vascular dementia |
| ICD-10 F02 | Dementia in other diseases classified elsewhere |
| ICD-10 F03 | Unspecified dementia |
| ICD-10 F05.1 | Delirium superimposed on dementia |
| ICD-10 F10.6 | Amnesic syndrome |
| ICD-10 G31.0 | Circumscribed brain atrophy |
| ICD-10 G31.1 | Senile degeneration of brain, not elsewhere classified |
| ICD-10 G31.8 | Other specified degenerative diseases of nervous system |
| ICD-10 G30 | Alzheimer's disease |
| ICD-10 I67.3 | Progressive vascular leukoencephalopathy |

Table S2. Risk of incident dementia below and above the change point of sunlight exposure in white participants.

| **Time spent in outdoor light** | **Model 1**  **(Age, Sex)** | | **Model 2**  **(+Education, Use of sun/UV protection，Employment status, Sleep duration, PM_2.5_)** | | **Model 3**  **(+Fracture history, Vitamin D supplement, hearing loss, smoking status, alcohol use, CVD, TPA, BMI)** | |
| --- | --- | --- | --- | --- | --- | --- |
|  | **HR (95%CI)** | **P Value** | **HR (95%CI)** | **P Value** | **HR (95%CI)** | **P Value** |
| **average** |  |  |  |  |  |  |
| 1.5 hours | reference |  | reference |  |  |  |
| below 1.5 hours (per 0.5 hour) | 1.290(1.126-1.479) | **<0.001** | 1.195(1.008-1.417) | **0.040** | 1.216 (1.012-1.461) | **0.037** |
| above 1.5 hours (per 0.5 hour) | 1.065(1.035-1.096) | **<0.001** | 1.077(1.037-1.119) | **<0.001** | 1.080 (1.037-1.127) | **<0.001** |
| **summer** |  |  |  |  |  |  |
| 2 hours | reference |  | reference |  |  |  |
| below 2 hours (per 1 hour) | 1.211(1.106-1.327) | **<0.001** | 1.147(1.022-1.287) | **0.020** | 1.152 (1.018-1.304) | **0.025** |
| above 2 hours (per 1 hour) | 1.026(1.005-1.047) | **0.013** | 1.032(1.005-1.060) | **0.018** | 1.030 (1.001-1.059) | **0.045** |
| **winter** |  |  |  |  |  |  |
| 1 hours | reference |  | reference |  |  |  |
| below 1 hours (per 1 hour) | 1.138(1.018-1.272) | **0.023** | 1.158(1.009-1.329) | **0.037** | 1.173 (1.012-1.359) | **0.034** |
| above 1 hours (per 1 hour) | 1.101(1.070-1.133) | **<0.001** | 1.119(1.077-1.163) | **<0.001** | 1.120 (1.075-1.168) | **<0.001** |

The results are derived from Cox proportional hazard regression models in three models. Bold indicates statistical significance (P value<0.05).

Abbreviation: HR, Hazard ratio; CI, Confidence interval; UV, Ultraviolet radiation; CVD, Cardiovascular disease; TPA, Total physical activity; BMI, Body mass index.

Table S3. Risk of incident dementia below and above the change point of sunlight exposure excluding participants less than three years of follow-up.

| **Time spent in outdoor light** | **Model 1**  **(Age, Sex)** | | **Model 2**  **(+Education, Skin color, Use of sun/UV protection, Employment status, Sleep duration, PM_2.5_)** | | **Model 3**  **(+Fracture history, Vitamin D supplement, hearing loss, smoking status, alcohol use, CVD, TPA, BMI)** | |
| --- | --- | --- | --- | --- | --- | --- |
|  | **HR (95%CI)** | **P Value** | **HR (95%CI)** | **P Value** | **HR (95%CI)** | **P Value** |
| **average** |  |  |  |  |  |  |
| 1.5 hours | reference |  | reference |  | reference |  |
| below 1.5 hours (per 0.5 hour) | 1.337 (1.179-1.517) | **<0.001** | 1.249 (1.067-1.463) | **0.006** | 1.276 (1.077-1.511) | **0.005** |
| above 1.5 hours (per 0.5 hour) | 1.078 (1.050-1.107) | **<0.001** | 1.075 (1.039-1.113) | **<0.001** | 1.077 (1.038-1.118) | **<0.001** |
| **summer** |  |  |  |  |  |  |
| 2 hours | reference |  | reference |  | reference |  |
| below 2 hours (per 1 hour) | 1.218 (1.120-1.326) | **<0.001** | 1.155 (1.037-1.286) | **0.009** | 1.158 (1.033-1.299) | **0.012** |
| above 2 hours (per 1 hour) | 1.037 (1.018-1.057) | **<0.001** | 1.034 (1.010-1.060) | **0.006** | 1.031 (1.004-1.057) | **0.022** |
| **winter** |  |  |  |  |  |  |
| 1 hours | reference |  | reference |  | reference |  |
| below 1 hours (per 1 hour) | 1.177 (1.063-1.303) | **0.002** | 1.178 (1.039-1.337) | **0.011** | 1.159 (1.012-1.327) | **0.032** |
| above 1 hours (per 1 hour) | 1.100 (1.072-1.130) | **<0.001** | 1.107 (1.069-1.146) | **<0.001** | 1.110 (1.069-1.153) | **<0.001** |

The results are derived from Cox proportional hazard regression models in three models. Bold indicates statistical significance (P value<0.05).

Abbreviation: HR, Hazard ratio; CI, Confidence interval; UV, Ultraviolet radiation; CVD, Cardiovascular disease; TPA, Total physical activity; BMI, Body mass index.

Table S4. Risk of incident dementia below and above the change point of sunlight exposure excluding participants less than ten years of follow-up.

| **Time spent in outdoor light** | **Model 1**  **(Age, Sex)** | | **Model 2**  **(+Education, Skin color, Use of sun/UV protection, Employment status, Sleep duration, PM_2.5_)** | | **Model 3**  **(+Fracture history, Vitamin D supplement, hearing loss, smoking status, alcohol use, CVD, TPA, BMI)** | |
| --- | --- | --- | --- | --- | --- | --- |
|  | **HR (95%CI)** | **P Value** | **HR (95%CI)** | **P Value** | **HR (95%CI)** | **P Value** |
| **average** |  |  |  |  |  |  |
| 1.5 hours | reference |  | reference |  |  |  |
| below 1.5 hours (per 0.5 hour) | 1.578(1.229-2.036) | **<0.001** | 1.601(1.185-2.163) | **0.002** | 1.614 (1.187-2.279) | **0.003** |
| above 1.5 hours (per 0.5 hour) | 1.124(1.068-1.182) | **<0.001** | 1.167(1.095-1.244) | **<0.001** | 1.137 (1.061-1.218) | **<0.001** |
| **summer** |  |  |  |  |  |  |
| 2 hours | reference |  | reference |  |  |  |
| below 2 hours (per 1 hour) | 1.346(1.139-1.591) | **<0.001** | 1.306(1.066-1.600) | **0.010** | 1.270 (1.020-1.583) | **0.033** |
| above 2 hours (per 1 hour) | 1.066(1.030-1.105) | **<0.001** | 1.088(1.041-1.136) | **<0.001** | 1.065 (1.016-1.116) | **0.008** |
| **winter** |  |  |  |  |  |  |
| 1 hours | reference |  | reference |  |  |  |
| below 1 hours (per 1 hour) | 1.324(1.082-1.620) | **0.007** | 1.364(1.069-1.740) | **0.013** | 1.334 (1.026-1.736) | **0.032** |
| above 1 hours (per 1 hour) | 1.118(1.063-1.175) | **<0.001** | 1.158(1.087-1.235) | **<0.001** | 1.140 (1.064-1.221) | **<0.001** |

The results are derived from Cox proportional hazard regression models in three models. Bold indicates statistical significance (P value<0.05).

Abbreviation: HR, Hazard ratio; CI, Confidence interval; UV, Ultraviolet radiation; CVD, Cardiovascular disease; TPA, Total physical activity; BMI, Body mass index.

Table S5. Risk of incident dementia below and above the change point of sunlight exposure in subgroup with complete covariates data (n=222479).

| **Time spent in outdoor light** | **Model 1**  **(Age, Sex)** | | **Model 2**  **(+Education, Skin color, Use of sun/UV protection, Employment status, Sleep duration, PM_2.5_)** | | **Model 3**  **(+Fracture history, Vitamin D supplement, hearing loss, smoking status, alcohol use, CVD, TPA, BMI)** | |
| --- | --- | --- | --- | --- | --- | --- |
|  | **HR (95%CI)** | **P Value** | **HR (95%CI)** | **P Value** | **HR (95%CI)** | **P Value** |
| **average** |  |  |  |  |  |  |
| 1.5 hours | reference |  | reference |  | reference |  |
| below 1.5 hours (per 0.5 hour) | 1.254 (1.048-1.499) | **0.013** | 1.298 (1.084-1.553) | **0.004** | 1.287 (1.094-1.515) | **0.002** |
| above 1.5 hours (per 0.5 hour) | 1.088 (1.048-1.130) | **<0.001** | 1.075 (1.034-1.116) | **<0.001** | 1.070 (1.031-1.110) | **<0.001** |
| **summer** |  |  |  |  |  |  |
| 2 hours | reference |  | reference |  | reference |  |
| below 2 hours (per 1 hour) | 1.120 (0.993-1.264) | 0.066 | 1.145 (1.014-1.293) | **0.028** | 1.193 (1.069-1.331) | **0.002** |
| above 2 hours (per 1 hour) | 1.039 (1.012-1.066) | **0.004** | 1.030 (1.003-1.057) | **0.028** | 1.026 (1.000-1.052) | **0.048** |
| **winter** |  |  |  |  |  |  |
| 1 hours | reference |  | reference |  | reference |  |
| below 1 hours (per 1 hour) | 1.139 (0.993-1.307) | 0.064 | 1.168 (1.017-1.341) | **0.028** | 1.184 (1.039-1.349) | **0.011** |
| above 1 hours (per 1 hour) | 1.108 (1.067-1.151) | **<0.001** | 1.103 (1.062-1.147) | **<0.001** | 1.106 (1.066-1.148) | **<0.001** |

The results are derived from Cox proportional hazard regression models in three models. Bold indicates statistical significance (P value<0.05).

Abbreviation: HR, Hazard ratio; CI, Confidence interval; UV, Ultraviolet radiation; CVD, Cardiovascular disease; TPA, Total physical activity; BMI, Body mass index.

Table S6. Risk of incident dementia below and above the change point of sunlight exposure in subgroups of age.

| **Time spent in outdoor light** | **<60 years old** | | | | **≥60 years old** | | | |
| --- | --- | --- | --- | --- | --- | --- | --- | --- |
|  | **Change point** | **P nonlinear** | **HR(95%CI)** | **P Value** | **Change point** | **P nonlinear** | **HR(95%CI)** | **P Value** |
| **average** |  |  |  |  |  |  |  |  |
| change point | 1.0 | 0.784 | reference |  | 1.9 | **0.027** | reference |  |
| below change point (per 0.5 hour) |  |  | 1.036 (0.715-1.502) | 0.853 |  |  | 1.344 (1.121-1.612) | **0.001** |
| above change point (per 0.5 hour) |  |  | 1.148 (1.051-1.254) | **0.002** |  |  | 1.054 (1.013-1.096) | **0.010** |
| **summer** |  |  |  |  |  |  |  |  |
| change point | 2.8 | 0.505 | reference |  | 2.9 | 0.151 | reference |  |
| below change point (per 1 hour) |  |  | 1.019 (0.801-1.297) | 0.877 |  |  | 1.239 (1.096-1.400) | **0.001** |
| above change point (per 1 hour) |  |  | 1.091 (1.021-1.167) | **0.010** |  |  | 1.016 (0.989-1.044) | 0.259 |
| **winter** |  |  |  |  |  |  |  |  |
| change point | NA | 0.562 | reference |  | 1.0 | **0.014** | reference |  |
| below change point (per 1 hour) |  |  | 0.998 (0.731-1.361) | 0.987 |  |  | 1.226 (1.062-1.417) | **0.005** |
| above change point (per 1 hour) |  |  | 1.176 (1.065-1.299) | **0.001** |  |  | 1.062 (1.026-1.100) | **0.001** |

The hazard ratio from Cox proportional hazard regression models adjusted for age, sex, education, skin color, use of sun/UV protection, employment status, sleep duration, and PM_2.5_, fracture history, Vitamin D supplement, hearing loss, smoking status, alcohol use, CVD, TPA, and BMI. Bold indicates statistical significance (P value<0.05).

Abbreviation: HR, Hazard ratio; CI, confidence interval; UV, Ultraviolet radiation; CVD, Cardiovascular disease; TPA, Total physical activity; BMI, Body mass index.

Table S7. Risk of incident dementia below and above the change point of sunlight exposure in subgroups of onset age.

| **Time spent in outdoor light** | **<65 years (Early onset)** | | | | **≥65 years (Lata onset)** | | | |
| --- | --- | --- | --- | --- | --- | --- | --- | --- |
|  | **Change point** | **P nonlinear** | **HR(95%CI)** | **P Value** | **Change point** | **P nonlinear** | **HR(95%CI)** | **P Value** |
| **average** |  |  |  |  |  |  |  |  |
| change point | 1.1 | 0.620 | reference |  | 1.8 | **0.031** | reference |  |
| below change point (per 0.5 hour) |  |  | 1.137 (0.732-1.766) | 0.568 |  |  | 1.304 (1.094-1.555) | **0.003** |
| above change point (per 0.5 hour) |  |  | 1.156 (1.029-1.299) | **0.015** |  |  | 1.063 (1.023-1.105) | **0.002** |
| **summer** |  |  |  |  |  |  |  |  |
| change point | 1.6 | 0.830 | reference |  | 2.5 | **0.041** | reference |  |
| below change point (per 1 hour) |  |  | 1.095 (0.823-1.455) | 0.534 |  |  | 1.205 (1.071-1.357) | **0.002** |
| above change point (per 1 hour) |  |  | 1.065 (0.985-1.151) | 0.114 |  |  | 1.023 (0.996-1.050) | 0.097 |
| **winter** |  |  |  |  |  |  |  |  |
| change point | 1.0 | 0.126 | reference |  | 1.0 | 0.054 | reference |  |
| below change point (per 1 hour) |  |  | 1.206 (0.848-1.716) | 0.298 |  |  | 1.174 (1.020-1.351) | **0.026** |
| above change point (per 1 hour) |  |  | 1.211 (1.079-1.358) | **0.001** |  |  | 1.068 (1.033-1.105) | **<0.001** |

The hazard ratio from Cox proportional hazard regression models adjusted for age, sex, education, skin color, use of sun/UV protection, employment status, sleep duration, and PM_2.5_, fracture history, Vitamin D supplement, hearing loss, smoking status, alcohol use, CVD, TPA, and BMI. Bold indicates statistical significance (P value<0.05).

Abbreviation: HR, Hazard ratio; CI, confidence interval; UV, Ultraviolet radiation; CVD, Cardiovascular disease; TPA, Total physical activity; BMI, Body mass index.

Table S8. Risk of incident dementia below and above the change point of sunlight exposure in sex subgroups.

| **Time spent in outdoor light** | **Male** | | | | **Female** | | | |
| --- | --- | --- | --- | --- | --- | --- | --- | --- |
|  | **Change point** | **P nonlinear** | **HR(95%CI)** | **P Value** | **Change point** | **P nonlinear** | **HR(95%CI)** | **P Value** |
| **average** |  |  |  |  |  |  |  |  |
| change point | 1.4 | 0.393 | reference |  | 1.6 | **0.009** | reference |  |
| below change point (per 0.5 hour) |  |  | 1.235 (0.972-1.569) | 0.085 |  |  | 1.340 (1.072-1.675) | **0.010** |
| above change point (per 0.5 hour) |  |  | 1.040 (0.992-1.091) | 0.106 |  |  | 1.123 (1.060-1.190) | **<0.001** |
| **summer** | 2.2 | 0.370 |  |  | 1.9 | **0.002** |  |  |
| change point |  |  | reference |  |  |  | reference |  |
| below change point (per 1 hour) |  |  | 1.131 (0.963-1.329) | 0.133 |  |  | 1.252 (1.078-1.455) | **0.003** |
| above change point (per 1 hour) |  |  | 1.019 (0.986-1.053) | 0.262 |  |  | 1.039 (0.999-1.081) | 0.058 |
| **winter** |  |  |  |  |  |  |  |  |
| change point | 1.0 | 0.762 | reference |  | 1.0 | **0.001** | reference |  |
| below change point (per 1 hour) |  |  | 1.036 (0.856-1.254) | 0.718 |  |  | 1.343 (1.121-1.609) | **0.001** |
| above change point (per 1 hour) |  |  | 1.069 (1.018-1.122) | **0.007** |  |  | 1.097 (1.045-1.152) | **<0.001** |

The hazard ratio from Cox proportional hazard regression models adjusted for age, education, skin color, use of sun/UV protection, employment status, sleep duration, and PM_2.5_, fracture history, Vitamin D supplement, hearing loss, smoking status, alcohol use, CVD, TPA, and BMI. Bold indicates statistical significance (P value<0.05).

Abbreviation: HR, Hazard ratio; CI, confidence interval; UV, Ultraviolet radiation; CVD, Cardiovascular disease; TPA, Total physical activity; BMI, Body mass index.

Table S9. Risk of incident dementia below and above the change point of sunlight exposure in subgroups of sleep duration.

| **Time spent in outdoor light** | **<7 hours per night** | | | | **7 hours per night** | | | **>7 hours per night** | | |
| --- | --- | --- | --- | --- | --- | --- | --- | --- | --- | --- |
|  | **P nonlinear** | **HR(95%CI)** | **P Value** | **P nonlinear** | | **HR(95%CI)** | **P Value** | **P nonlinear** | **HR(95%CI)** | **P Value** |
| **average** |  |  |  |  | |  |  |  |  |  |
| 1.5 hours | **0.025** | reference |  | 0.890 | | reference |  | **0.007** | reference |  |
| below 1.5 hours (per 0.5 hour) |  | 1.301 (0.980-1.726) | 0.069 |  | | 1.087 (0.890-1.328) | 0.411 |  | 1.426 (1.138-1.788) | **0.002** |
| above 1.5 hours (per 0.5 hour) |  | 1.127 (1.060-1.199) | **<0.001** |  | | 1.025 (0.986-1.066) | 0.218 |  | 1.032 (0.986-1.080) | 0.174 |
| **summer** |  |  |  |  | |  |  |  |  |  |
| 2 hours | **0.002** | reference |  | 0.051 | | reference |  | **0.008** | reference |  |
| below 2 hours (per 1 hour) |  | 1.143 (0.968-1.350) | 0.116 |  | | 1.183 (1.044-1.339) | **0.008** |  | 1.418 (1.231-1.633) | **<0.001** |
| above 2 hours (per 1 hour) |  | 1.060 (1.018-1.104) | **0.005** |  | | 1.015 (0.989-1.042) | 0.267 |  | 1.015 (0.985-1.046) | 0.323 |
| **winter** |  |  |  |  | |  |  |  |  |  |
| 1 hour | 0.308 | reference |  | **0.032** | | reference |  | 0.303 | reference |  |
| below 1 hour (per 1 hour) |  | 1.132 (0.910-1.408) | 0.267 |  | | 1.164 (1.009-1.343) | **0.037** |  | 1.228 (1.042-1.448) | **0.014** |
| above 1 hour (per 1 hour) |  | 1.090 (1.031-1.154) | **0.003** |  | | 1.082 (1.043-1.123) | **<0.001** |  | 1.087 (1.041-1.135) | **<0.001** |

The hazard ratio from Cox proportional hazard regression models adjusted for age, sex, education, skin color, use of sun/UV protection, employment status, PM_2.5_, fracture history, Vitamin D supplement, hearing loss, smoking status, alcohol use, CVD, TPA, and BMI. Bold indicates statistical significance (P value<0.05).

Abbreviation: HR, Hazard ratio; CI, confidence interval; UV, Ultraviolet radiation; CVD, Cardiovascular disease; TPA, Total physical activity; BMI, Body mass index.
